# Distance- and blended-learning in global health research: potentials and challenges

**DOI:** 10.3402/gha.v9.33429

**Published:** 2016-10-06

**Authors:** Henry Lucas, John Kinsman

**Affiliations:** 1 Health and Nutrition, Institute of Development Studies, University of Sussex, Brighton BN1 9RE, UK; 2 Division of Epidemiology and Global Health, Department of Public Health and Clinical Medicine, Umeå University, 901 87 Umeå, Sweden

It has been argued that in every country, ‘social, educational, technological, and economic development fundamentally depends on the advancement of science through research … and [it] benefits from having a … network of actors engaged in promoting and using scientific research’ (1), p. 26). This applies in particular to life sciences research in low- and middle-income countries (LMICs), given that many such countries face the heaviest burdens of disease. However, Langer et al. (2) lamented in 2004, that ‘In the fields of medicine and public health … papers where researchers from developing countries are the sole authors represent a very low proportion of published manuscripts’ (p. 802). The reasons identified for this include: poor access to scientific literature, poor participation in publication-related decision-making processes, and the bias of journals. Much has changed since then, with a dramatic growth in the number of journals addressing public health concerns, many of which are based in LMICs or which include LMIC researchers on their editorial boards. There have been substantial initiatives, most notably Hinari (3), to provide LMIC researchers with access to the scientific literature. However, though the number of LMIC publications has increased substantially, a recent publication (4) found no LMIC in the top forty countries in terms of publications per capita.

The other challenges identified by Langer et al. (2) can be seen as relating to inadequate research capacity, in large part resulting from limited resources in terms of both money and time. Younger academics face significant difficulties both in competing for support from international funding agencies and in persuading those agencies of the fund-management abilities of their institutions. Many will also have substantial teaching commitments which impose severe time constraints. Rather than pursuing their personal research interests, many will opt for the easier option of joining a project led by researchers from high-income counties (5). Attempts to build LMIC capacity are further constrained by the small number of senior research staff with proven track records available to act as mentors. Such individuals are typically in great demand. Some may join the ‘brain drain’ (6) to high-income countries, while others may contract out their teaching commitments in order to work more lucratively with international agencies. Students are often aware of this situation and the most able may seek improved educational opportunities overseas, further depleting the local research network.

For some time, it has been suggested that developments in information and communications technologies (ICTs) – such as increased access to the Internet, wide-spread ownership of mobile smart phones, and declining costs of computer hardware and software – could be used to transform the traditional approach to medical school education in high-income countries (7). More recently, similar applications have been advocated as a way to break through the human resource barriers limiting the pace of research capacity building in LMICs (8, 9). It has been suggested that ICTs can provide the means to deliver advanced, high quality instruction to the very substantial number of individuals in LMICs who have both the motivation and capacity to benefit, but who face insuperable financial or social barriers in terms of gaining access to the traditional courses offered by universities and colleges (10).

Evidence for this includes the astonishingly rapid growth of massive open online courses (MOOCs). By 2013, Coursera, the largest provider of MOOCs, announced that it had enrolled over five million students on 500 courses and that it had agreed partnership arrangements with over 100 academic institutions around the world (11). While identification and classification of the courses on offer can be problematic, one study estimated that 98 free courses on human health and medicine, many provided by highly regarded US medical colleges, were available from 12 MOOC providers in 2013 (12). The success of this model would seem to suggest that, in principle, e-learning –‘the use of digital or electronic technologies and materials to support teaching and learning’ (13) – could dramatically increase the number of students able to access tertiary education. This could be achieved using a combination of self-tuition via distance learning as with the MOOCs, virtual classrooms which allow scope for real-time feedback, or, where possible, blended learning which involves using online resources to complement face-to-face instruction.

Such enthusiasm needs to be tempered by considering some of the potential hurdles which will confront those attempting to follow the e-learning route. One obvious issue relates to the enormous gap in terms of the provision of online resources. Of the 98 courses mentioned above, only two were offered by LMIC countries (China and The West Indies). The 2013 Coursera announcement also indicated that only a tiny proportion of participants were from the African continent. Most MOOC courses rely on video lectures which require a very fast broadband link (14). However, in many LMIC academic institutions, even the much less demanding and much less compelling format of ‘voice-over-PowerPoint’ presentations will test available bandwidth limitations (15). Many institutions will also struggle to allocate the necessary funds to purchase the necessary hardware and software required to establish an effective and reliable system for accessing online materials. Further, they may be very concerned about the longer term implications in terms of the maintenance, upgrading, and need for routine replacement of key components. Even if the finance is available, serious human resource issues may still be faced. In many LMICs, highly skilled ICT staff are in very short supply, and may not easily adapt to the time-consuming working relationships with academic staff typically required to transform existing courses into e-learning formats (16).

As evidenced by a number of the articles in this Special Issue, there also remain institutional and personal barriers to educational strategies based on e-learning. Established universities and colleges in many LMICs are understandably concerned to maintain their, often hard won, reputation for academic rigour and excellence. They will likely regard their existing statutes, regulations, and protocols as key to that reputation; changing, for example, the number of contact hours required to complete a course in order to accommodate the requirements of a blended-learning approach may entail the consent of one or more high-level committees, a process that can be drawn-out and complex. Novel processes for gaining course credits may also raise critical issues, relating, for example, to plagiarism or prohibitions on collaborative working (17). The inability to gain a recognised qualification is another important reason often cited by potential students for not enrolling on some courses offered by MOOC providers (14). Finally, it must be acknowledged that many excellent lecturers and highly capable students may simply not find e-learning options attractive. Senior academics will often see little value in allocating valuable time away from their primary teaching and research interests to acquire the necessary skills. They may be convinced that fact-to-face discussion is the only effective, or at least the best way to lead students to an in-depth understanding of complex concepts. Similarly, while some students may welcome the opportunity to proceed at their own pace, free from the intimidating oversight of their teachers, others, especially those who have progressed through traditional, formal education systems, can find great difficulty in adapting to an unfamiliar and unstructured learning environment.

In this Special Issue, Atkins et al. (18) present a detailed documentary review to describe the aims and nature of two capacity building projects that have explored the possibilities for e-learning provided by advances in ICT. The projects were delivered from 2011 to 2015 under the heading ‘African/Asian Research Capacity Development’ (ARCADE). The first focused on Health Systems and Services Research (HSSR) in Southern Africa, with a partnership involving Malawi, Norway, Sweden South Africa, Tanzania, Uganda, and the United Kingdom, and the second focused on Research on Social Determinants of Health (RSDH) in Asia, with partners from China, Finland, India, Oman, South Africa, Sweden, the United Kingdom, and Vietnam. The projects involved collaboration between universities in each of these countries that involved not only joint development and delivery of 55 blended and distance-learning courses, attended by 920 masters and doctoral students of whom around 50% were female, but also capacity building in research financing, grant management, and the dissemination of findings. Some 60 collaborative research proposals were also developed by project participants. The main challenge in the work was identified as the relatively limited previous engagement with e-learning at both the institutional and personal levels in most of the Southern partner universities. Over the course of the projects, there was an increasing appreciation of the possible benefits to be gained, but the process of first engaging with the technology and then identifying and overcoming often unanticipated obstacles allowed limited time for course design and implementation.

The second article by Färnman et al. (19) discusses the lessons which can be drawn from the ARCADE projects in terms of the future development of North–South research partnerships. The research for the article involved key informant interviews with 16 participants from 12 of the partner institutions, combined with a review of the project reports. As above, this article stresses the importance of ensuring that new e-learning courses meet existing university requirements, including the award of course credits. There was also a suggestion that future exercises of this nature should be more closely tailored to the diverse contexts of each partner institution, for example, in terms of technical and human resource constraints.

In the third article, Atkins et al. (20) consider one of the major components of the ARCADE projects: the collaborative design and implementation of ‘blended-learning courses’, which combine face-to-face instruction with a range of complementary e-learning activities. The article focuses on student experiences of these courses and is based on an online questionnaire survey which was completed by 82 of the 118 students in participating African, Asian, and European partner institutions. A regression analysis showed that while there was significant variation in experiences between courses, the common factors influencing student perceptions of the online components were the lack or otherwise of technical problems, and the extent to which there were opportunities for feedback and discussion.

The fourth article, by Atkins et al. (21), is focused on another component of the ARCADE projects, a series of ‘research clinics’. These comprised seven online webinars, each based on a relatively junior researcher's presentation of their research proposal or paper, and attended online by both graduate students and academic staff from partner universities. The article is based on analysis of interviews with the presenting researcher and a sample of other participants. The webinars are said to have been well attended and organised, with the junior researchers enthusiastic about to the potential improvements that could be made to their proposals based on the suggestions provided.

In a complementary article, Protsiv and Atkins (22) consider the experiences of 11 lecturers involved in designing and delivering blended-learning courses in universities in India, South Africa, Sweden, and Uganda. The article is based on a series of key informant interviews. The main findings contrast the enthusiasm with which the lecturers embraced the possibilities of the technology with their frustration at the technical and logistical barriers which had to be overcome. These related to such factors as the limited capacity of the local Internet infrastructure, the need to use unfamiliar software, and what they regarded as limited institutional support. The informants also spoke of the unexpectedly high time investment required by this teaching approach, even in transforming an existing course into the blended format.

Finally, Kumpu et al. (23) address the relatively unexplored issue of the economics of providing such collaborative blended-learning courses, using as a case study a course on randomised controlled trials run jointly across three universities in South Africa, Sweden, and Uganda. The course was led by Stellenbosch University and was joined by students at Karolinska Institutet and Makerere University. In all three universities, cost estimates were derived using activity-based costing with an ingredients approach; the costs of the same course delivered in a traditional manner were also estimated for Stellenbosch. Learning outcomes in the three universities were assessed using course grades. Given the findings presented above concerning the time required to prepare and deliver blended courses for the first time, it is perhaps not surprising that the cost was found to be considerably higher than that for the course taught using traditional methods. The main component, staff costs, was estimated to be three times higher, while learning outcomes appear similar to those obtained for the traditional course.

Collectively, these papers provide an important set of insights into both the opportunities and the challenges that accompany multi-institute, blended-learning health research training. This educational approach clearly presents huge possibilities for a genuinely global exchange of skills and capacities, and it also represents an invaluable supplement to traditional, face-to-face university-based learning as well as to the MOOCs that provide free, but usually unaccredited, education to anyone with an Internet connection. However, there remain significant challenges, not least of which are cost, inadequate ICT infrastructure in some areas, and the time and effort required by lecturers to develop and present the courses in this format. The lessons learned by the ARCADE projects that are presented in this Special Issue provide a good basis for advancing blended-learning health research training as an attractive and sustainable educational approach.

*Henry Lucas* Health and Nutrition Institute of Development Studies University of Sussex Brighton BN1 9RE UK *John Kinsman* Division of Epidemiology and Global Health Department of Public Health and Clinical Medicine Umeå University, 901 87 Umeå Sweden
